# Enlargements of the Spleen—IX

**Published:** 1908-10-03

**Authors:** 


### ENLARGEMENTS OF THE SPLEEN—IX.

### SPLENIC ENLARGEMENTS IN WHICH THE BLOOD CHANGES ARE NOT DISTINCTIVE.

*Rickets; Congenital Syphilis; Pseudo-leuchæmia Infantum.*—These three conditions may be taken together in connection with the enlargement of the spleen that may be associated with them in childhood; for although there are many observers who regard the splenic lesions in the three conditions as quite separate and distinct, there is no doubt that there is a greater likelihood of splenic enlargement when congenital syphilis and rickets coincide than when either occurs alone; and there are some who believe that pseudo-leuchæmia infantum is really an exaggerated degree of the congenital syphilitic cachexia and enlargement of the spleen—although this is a point upon which there have been great discussions without any decisive result.

In rickets the spleen is often palpable without being actually enlarged; owing to the diminished size of the thorax and the eversion of the lower ribs, in some cases the normal organ may come below the costal margin, and thus appear to be bigger than it really is. Sometimes, however, it is undoubtedly increased in size, either slightly or moderately. The diagnosis depends upon the co-existence of other signs of rickets. It is most likely to be confused with congenital syphilis.

In some cases of rickets the blood is normal. In others, however, it is of the chlorotic type, the red corpuscles being slightly diminished and the hæmoglobin considerably so, the colour index being something like 0.7 or less. The leucocytes may be slightly increased, up to 10,000 per cub. mm. for example; they seldom reach 30,000. The differential leucocyte count nearly always gives a relatively high proportion of small lymphocytes—40 per cent. or more—but this is the case in many other children's affections; the lymphocytes are normally more numerous in the young than in adults.

The enlarged spleen of rickets nearly always assumes its normal size as the patient grows up, differing in this respect from the condition in some cases of congenital syphilis. In the latter the splenic tumour may persist into later life, when the diagnosis will be given by finding pegged or notched permanent teeth, interstitial keratitis or corneal opacities, or radiating linear scars about the month or anus. There may be a chlorotic type of anæmia, but there is nothing distinctive about the blood count.

*Pseudo-leuchæmia infantum* was first described by von Jaksch in 1889. It affects infants and young children from six months to four years of age. Out of twenty cases collected by Monti and Berggrum evidence of rickets was found in sixteen, congenital syphilis, chronic tuberculosis, and chronic intestinal catarrh also being regarded as causes in some cases.

The disease is characterised by very grave anæmia, great enlargement of the spleen, moderate increase in the size of the liver, and protuberance of the abdomen. The blood counts show great departure from the normal, and yet there is nothing absolutely pathognomonic about them. The leucocytes may not be increased at all, but occasionally there is a rise to as many as 15,000 or 20,000 per cub. mm. The differential leucocyte count exhibits a relative increase in the small lymphocytes, and both basophile cells and myelocytes may be present. The red corpuscles are diminished to a variable extent—in some cases they may be observed to fall steadily week by week until they reach as low a figure as 1,000,000 per cub. mm. or less. The patient by this time looks absolutely pallid; the hæmoglobin fails even more rapidly than does the number of red cells, and the colour index may be as low as 0.3. Poikilocytosis becomes a marked feature of the film, and nucleated red cells, some of which show mitosis, may be as numerous as they are in any other severe anæmia whatever. It is a noteworthy fact, however, that even when this severe degree of anæmia has been reached the prognosis is not absolutely bad; under treatment by arsenic, iron, and mercury the most wonderful rallying power is exhibited in some cases. An infant who has been upon the verge of death for months, with an enormous spleen, a red count of less than 1,000,000 per cub. mm., and many normoblasts, and megaloblasts, may suddenly improve, his spleen may become much smaller, and he may attain 5,000,000 red corpuscles per cub. mm., with nearly a full percentage of hæmoglobin. When this reaction has occurred it is quite possible that the child will remain well; relapses are possible, but there is not the same certainty about them that there is in pernicious anæmia; the latter never occurs in children.

*Splenomegalic cirrhosis.*—In all cases of cirrhosis of the liver the spleen is more or less enlarged at the post-mortem examination, although it may not have been palpable during life. In some cases, on the other hand, the splenic enlargement is enormous, so that it attracts the chief attention both of the patient and of the medical man. When the patient is an adult, the case is usually diagnosed as splenic anæmia in the first instance, only later being recognised as cirrhosis—a point which will be referred to under the heading of Banti's disease in our next and concluding article. When the patient is younger, on the other hand, the lesion is termed splenomegalic cirrhosis, and, as a rule, a marked feature of the case is chronic jaundice. The blood condition is entirely negative—there may be some degree of anæmia of the chlorotic type, but nothing more. If, however, with these negative blood changes, there is a huge spleen and chronic jaundice in a boy or girl, the probability is that the condition is one of cirrhosis. The nature of the latter is obscure. In a few cases alcoholism has begun even at this age. In others, however, no alcoholism can be proved, and it is a remarkable fact that there is sometimes a family tendency to the trouble—parent and child may have it, or several children in the same family may be sufferers from the same complaint. Congenital syphilis is sometimes regarded as a cause.

*(To be concluded.)*

